# Effect of Crown Height Space and Abutment Configuration on the Maximum Recorded Load of Implant-Supported Monolithic Zirconia Crown Assemblies

**DOI:** 10.3390/ma19214683

**Published:** 2026-11-03

**Authors:** Özay Özsoy, Melek Nur Ürün, Şeyda Bektaş, Serdar Polat

**Affiliations:** Department of Prosthodontics, Faculty of Dentistry, Gazi University, Ankara 06490, Turkey; urunmeleknur@gmail.com (M.N.Ü.); seyda.bektas1234@gmail.com (Ş.B.); drserdarpolat@gmail.com (S.P.)

**Keywords:** crown height, Ti-base, multi-unit abutment, monolithic zirconia, maximum recorded load, screw-retained restoration, implant-supported restoration

## Abstract

This in vitro study evaluated the effects of crown height space (CHS) and abutment configuration on maximum recorded load and described post-test damage in implant-supported monolithic zirconia crown assemblies. Sixty specimens were assigned to six groups (*n* = 10) combining Ti-base or multi-unit configurations with nominal CHS levels of approximately 13.5, 15.5, or 17.5 mm. After 200,000 thermomechanical aging cycles at 50 N with thermal cycling between 5 °C and 55 °C, specimens underwent eccentric vertical loading on the buccal incline of the functional cusp using a 4 mm spherical tip at 1 mm/min. The primary outcome was maximum recorded load (global peak load), not the onset of irreversible damage. Two-way ANOVA indicated a CHS effect (*p* = 0.012), but no significant effect of abutment configuration (*p* = 0.128) or interaction (*p* = 0.627). The CHS effect persisted in the HC3 analysis (*p* = 0.043). The lowest versus highest CHS comparison remained significant in heteroscedasticity-sensitive analyses; the lowest-versus-intermediate comparison depended on analytical approach. Four zirconia crown fractures occurred in the lowest-CHS Ti-base group; other post-test damage predominantly involved prosthetic connection components. Within these laboratory conditions, CHS influenced assembly-level maximum recorded load without establishing a continuous or linear relationship.

## 1. Introduction

Alveolar bone resorption following tooth loss may increase the interocclusal space available for implant-supported prosthetic rehabilitation, thereby requiring restorations with greater vertical dimensions. This increased vertical space may influence not only prosthetic design but also the biomechanical behavior of the implant–abutment–restoration complex. Crown height space (CHS) is defined as the vertical distance from the alveolar bone crest to the occlusal plane in the posterior region and represents an important parameter in the biomechanical assessment of implant-supported restorations. Increased CHS may increase biomechanical demands, particularly in the presence of lateral or oblique force components, potentially generating greater stresses at the implant–abutment connection and in the peri-implant bone [1].

To evaluate the biomechanical effects of increased vertical space in implant-supported restorations, various geometric parameters, including the crown-to-implant ratio (CRI) and crown height space (CHS), have been used. Although CRI describes the relationship between restorative crown height and the bone-supported length of the implant, the available evidence regarding its influence on clinical outcomes remains inconsistent [2,3]. Systematic reviews have reported that an increased CRI is not directly or consistently associated with implant survival, marginal bone loss, or prosthetic complications [3]. In contrast, CHS is an absolute vertical measurement rather than a ratio, offering a complementary biomechanical perspective. Finite element studies have demonstrated that increasing crown height may alter stress distribution within the implant–abutment complex, prosthetic components, and peri-implant structures [4,5]. Experimental studies have also reported that increased restorative height may influence the mechanical resistance and stability of the implant–abutment system [5,6,7]. These findings highlight the importance of considering not only the crown-to-implant ratio but also the absolute vertical height of the restoration when evaluating the mechanical behavior of implant-supported restorations. The selected CHS levels were intended to represent progressively increasing vertical restorative space that may be encountered following alveolar ridge resorption. Three conditions were created using 2-mm increments in the restorative portion to provide a standardized and systematic comparison across low-, intermediate-, and high-CHS conditions. When the simulated crestal bone level and the corresponding gingival abutment heights were taken into account, these conditions resulted in nominal CHS levels of approximately 13.5, 15.5, and 17.5 mm. Previous experimental studies have evaluated crown or suprastructure heights ranging from approximately 7.5 to 18 mm, including 8, 10, 12, and 14 mm; 10, 14, and 18 mm; and 7.5, 10.5, and 13.5 mm configurations. Thus, the selected CHS levels provided a controlled 2-mm progression while remaining within the range investigated in previous biomechanical studies [1,6,7,8].

With advances in CAD/CAM technologies, monolithic zirconia has become one of the most widely used restorative materials for implant-supported fixed restorations because of its favorable mechanical properties and biocompatibility [9]. The prosthetic connection design used in implant-supported zirconia restorations is another important factor that may influence the mechanical performance of the restoration. Ti-base components provide a metallic interface between the ceramic restoration and the implant, thereby combining the mechanical advantages of a titanium connection with CAD/CAM-fabricated ceramic restorations [10]. However, the geometry and height of the Ti-base have also been reported to influence the mechanical stability of implant-supported restorations [11].

Multi-unit abutments, in contrast, shift the prosthetic connection from the implant level to the abutment level, allowing the restoration to be connected to the implant through a different component and connection configuration. Accordingly, Ti-base- and multi-unit-based restorations represent distinct mechanical configurations in terms of connection level, number of components, and the manner in which loads are transferred through the implant–prosthetic complex. Laboratory studies have demonstrated that differences in titanium base design and implant–abutment connection configuration may affect load-bearing capacity, deformation, and failure modes [11,12]. Studies comparing Ti-base and other abutment configurations in monolithic zirconia restorations further support the concept that mechanical performance may depend not only on the restorative material itself but also on how the restoration is connected to the implant system [13,14].

Experimental studies evaluating different vertical heights have reported that this geometric factor may affect the load-bearing capacity, connection stability, and failure behavior of the implant–abutment complex [6,7,8]. However, a substantial proportion of the available studies have focused on different implant connection designs or restorative materials, and data directly comparing the effect of CHS across distinct prosthetic connection configurations, such as Ti-base and multi-unit systems, within the same experimental model remain limited. Therefore, evaluating different CHS levels in Ti-base and multi-unit configurations using the same implant system and monolithic zirconia material allows a direct comparison of the effects of CHS and abutment configuration on the mechanical behavior of the implant–abutment–crown complex.

The aim of this study was to evaluate the effects of three nominal CHS levels (approximately 13.5, 15.5, and 17.5 mm) and two different abutment configurations (Ti-base and multi-unit) on the maximum recorded load and failure patterns of implant-supported monolithic zirconia crown assemblies. The first null hypothesis was that abutment configuration would have no significant effect on maximum recorded load; the second was that no significant difference in maximum recorded load would be observed among the CHS levels; and the third was that there would be no significant interaction between CHS and abutment configuration.

## 2. Materials and Methods

This in vitro study evaluated the effects of abutment configuration and crown height space (CHS) on the maximum recorded load (global peak load) of implant-supported monolithic zirconia crown assemblies. Sixty NucleOSS^®^ dental implants (TRQ 4012; Şanlılar Tıbbi Cihazlar, İzmir, Türkiye), measuring 4 mm in diameter and 12 mm in length, were used. All specimens were prepared using a standardized model to minimize variations in implant position, axial orientation, and embedding depth.

Two prosthetic connection configurations were evaluated: Ti-base and multi-unit. Both configurations incorporated HEX-indexed, anti-rotational components appropriate for unsplinted single-crown restorations.

In the Ti-base configuration, a NucleOSS CAD/CAM Ti-base (T6 32806) with a gingival height of 2.4 mm and a HEX connection was used for single-tooth restorations. The monolithic zirconia crowns were cemented directly onto these components. The prosthetic screws were tightened to 25 N·cm using a manufacturer-supplied calibrated manual torque wrench and ratchet, in accordance with the manufacturer’s recommendations.

In the multi-unit configuration, straight 0° multi-unit abutments (T6 32414), each with a gingival height of 2.5 mm and a HEX connection, were attached to the implants and tightened to 25 N·cm. A short, HEX-indexed multi-unit CAD/CAM Ti-base (T6 32814) was then connected to each abutment, and the monolithic zirconia crown was cemented onto this titanium component. The prosthetic screw connecting the CAD/CAM Ti-base–crown assembly to the multi-unit abutment was tightened to 15 N·cm using the same torque-control system.

Both configurations incorporated Ti-base components with a nominal height of 4.5 mm, which remained constant across all three CHS levels. According to the manufacturer, the prosthetic platform diameters were 4.5 mm for the implant-level Ti-base (T6 32806) and 4.8 mm for the multi-unit CAD/CAM Ti-base (T6 32814). All screw connections were retightened once to their initial torque values 10 min after the first tightening, before thermomechanical aging. No further retightening was performed after aging. Before aging, the screw-access channels were sealed with polytetrafluoroethylene (PTFE) tape and composite resin.

CHS was defined as the vertical distance from the simulated crestal bone level to the occlusal plane, measured parallel to the implant long axis. The embedding/support plane was positioned 3 mm apical to the implant platform and served as the simulated crestal bone reference. Total CHS was calculated by combining this 3-mm distance, the gingival height of the prosthetic component, and the vertical dimension of the restoration.

**Figure 1:**
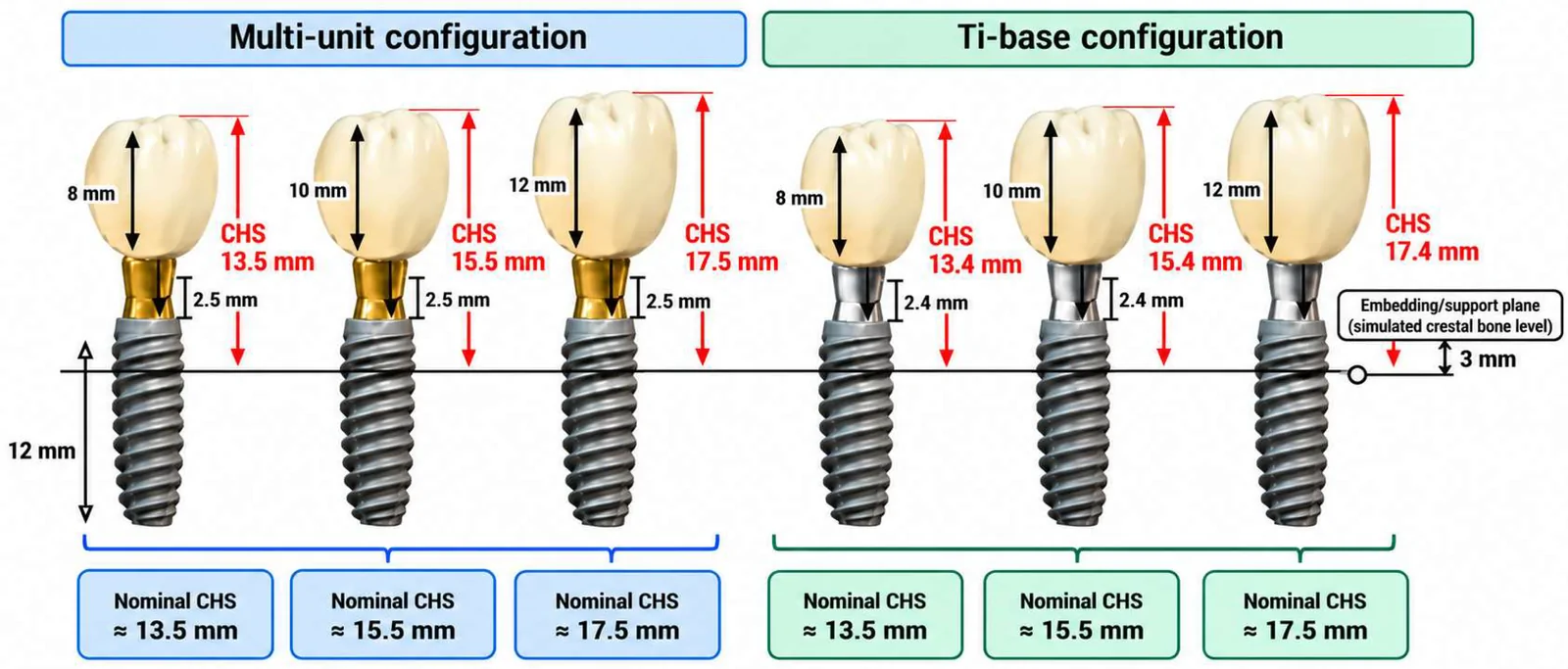
Schematic representation of the three nominal crown height space (CHS) levels (approximately 13.5, 15.5, and 17.5 mm) evaluated in the Ti-base and multi-unit configurations. CHS was measured parallel to the implant long axis from the simulated crestal bone level (embedding/support plane positioned 3 mm apical to the implant platform) to the occlusal plane. The actual CHS values differed by 0.1 mm between configurations owing to differences in gingival component height.

Three nominal CHS levels of approximately 13.5, 15.5, and 17.5 mm were evaluated (Figure 1). Because the gingival heights of the Ti-base and multi-unit components were 2.4 and 2.5 mm, respectively, the actual CHS values differed by 0.1 mm between corresponding configurations, with slightly lower values in the Ti-base groups. The same nominal CHS designations were therefore used for both configurations to ensure consistent group classification and statistical analysis. These designations refer to the original experimental groups and do not represent additional specimens or experimental conditions. Accordingly, the 60 specimens were allocated to six groups in a 2 × 3 factorial design (*n* = 10 per group). No a priori power analysis was performed.

A single master gypsum model was obtained from an impression and used as the reference for all restorative designs. After placement of the appropriate scan bodies, the model was digitized using a BLZ LS-100 laboratory scanner (Redon Teknoloji, Istanbul, Türkiye). All crowns were designed from the resulting digital model using exocad DentalCAD 3.2 Elefsina software (exocad GmbH, Darmstadt, Germany). This standardized workflow provided a common reference for implant/analog positioning, implant-axis orientation, and screw-access channel placement across the experimental groups.

The screw-access channel was positioned as centrally as possible within each zirconia restoration while maintaining adequate surrounding ceramic support. The three CHS conditions were established by increasing the vertical dimension of the restoration along the implant long axis without proportionally scaling the entire crown. Comparable mandibular first-molar morphology, cusp anatomy, and cusp inclinations were targeted across all groups. The intended loading region was the buccal incline of the functional cusp, and the cement space was standardized at 35 µm.

Archived exocad design-view files for all six experimental groups were retained and are provided as Supplementary File S3. These files document the digital restoration designs for the Ti-base and multi-unit configurations at the three nominal CHS levels (approximately 13.5, 15.5, and 17.5 mm). They allow visual inspection of the occlusal morphology and the zirconia surrounding the screw-access channel. However, the archived digital designs do not establish the exact loading contact coordinates during mechanical testing.

The restorations were milled from 16-mm-thick Explore Functional multilayer zirconia discs (Upcera Dental, Shenzhen, China), incorporating a 3Y-TZP/4Y-PSZ compositional gradient, using a Redon Hybrid CAD/CAM milling system (Redon Teknoloji, Istanbul, Türkiye). During nesting, the crowns were positioned centrally within the disc thickness, with their occlusal surfaces oriented toward the upper layers and their cervical portions toward the lower layers. Ten crowns per experimental group were fabricated using the corresponding CAD design.

Following milling, the crowns were sintered in a Redon Rapid furnace (Redon Teknoloji, Istanbul, Türkiye) using the manufacturer’s recommended sintering program. The temperature was increased to 1150 °C at a heating rate of 8 °C/min and maintained for 30 min. It was then increased to 1300 °C at 2 °C/min and subsequently to 1480 °C at 4 °C/min. After holding at 1480 °C for 120 min, the temperature was reduced to 800 °C at 8 °C/min, followed by natural cooling.

All zirconia crowns were cemented using a self-adhesive dual-cure resin cement (seT PP, SDI Limited, Bayswater, Victoria, Australia). Before cementation, the zirconia intaglio surfaces were airborne-particle abraded with 50-µm aluminum oxide (Al_2_O_3_) particles at a pressure of 0.2 MPa, from a distance of approximately 5 mm, for 15 s. The treated surfaces were subsequently cleaned with pressurized steam for 15 s. No separate primer was applied to the zirconia or titanium bonding surfaces.

The zirconia crowns were cemented directly onto the implant-level Ti-base components in the Ti-base groups and onto the corresponding CAD/CAM titanium components in the multi-unit groups. Following seating, the resin cement was light-cured for 20 s from each accessible surface. The same zirconia surface-conditioning, cementation, and polymerization procedures were applied across all experimental groups.

Before thermomechanical aging, the implants were embedded in a cold-cure autopolymerizing acrylic resin (İntegra Cold-Cure Pink Acrylic, İntegra, İzmir, Türkiye). All implants were positioned vertically at a standardized embedding depth, with the embedding/support plane located 3 mm apical to the implant platform.

All specimens underwent thermomechanical aging using a MOD-DENTAL chewing simulator (Esetron Smart Robot Technologies, Ankara, Türkiye) before static loading. Mechanical aging was performed using a 4-mm-diameter spherical stainless-steel antagonist under a 50-N load at a frequency of 1.53 Hz for 200,000 cycles. The simulator was programmed to deliver a 6-mm vertical stroke at a movement speed of 27 mm/s, without a horizontal sliding component. The 50-N load was selected to simulate masticatory loading conditions and was consistent with values used in previous thermomechanical aging studies of implant-supported zirconia restorations [13].

Thermal cycling between 5 °C and 55 °C was performed simultaneously with mechanical loading [13]. Temperature changes were achieved using the simulator’s integrated hot- and cold-water circulation system, with a programmed dwell time of 60 s at each temperature. The specimens remained fixed throughout the procedure, without being transferred between separate temperature-controlled baths. However, the exact number of completed thermal cycles and the duration of temperature transitions could not be retrospectively verified from the available records.

**Figure 2:**
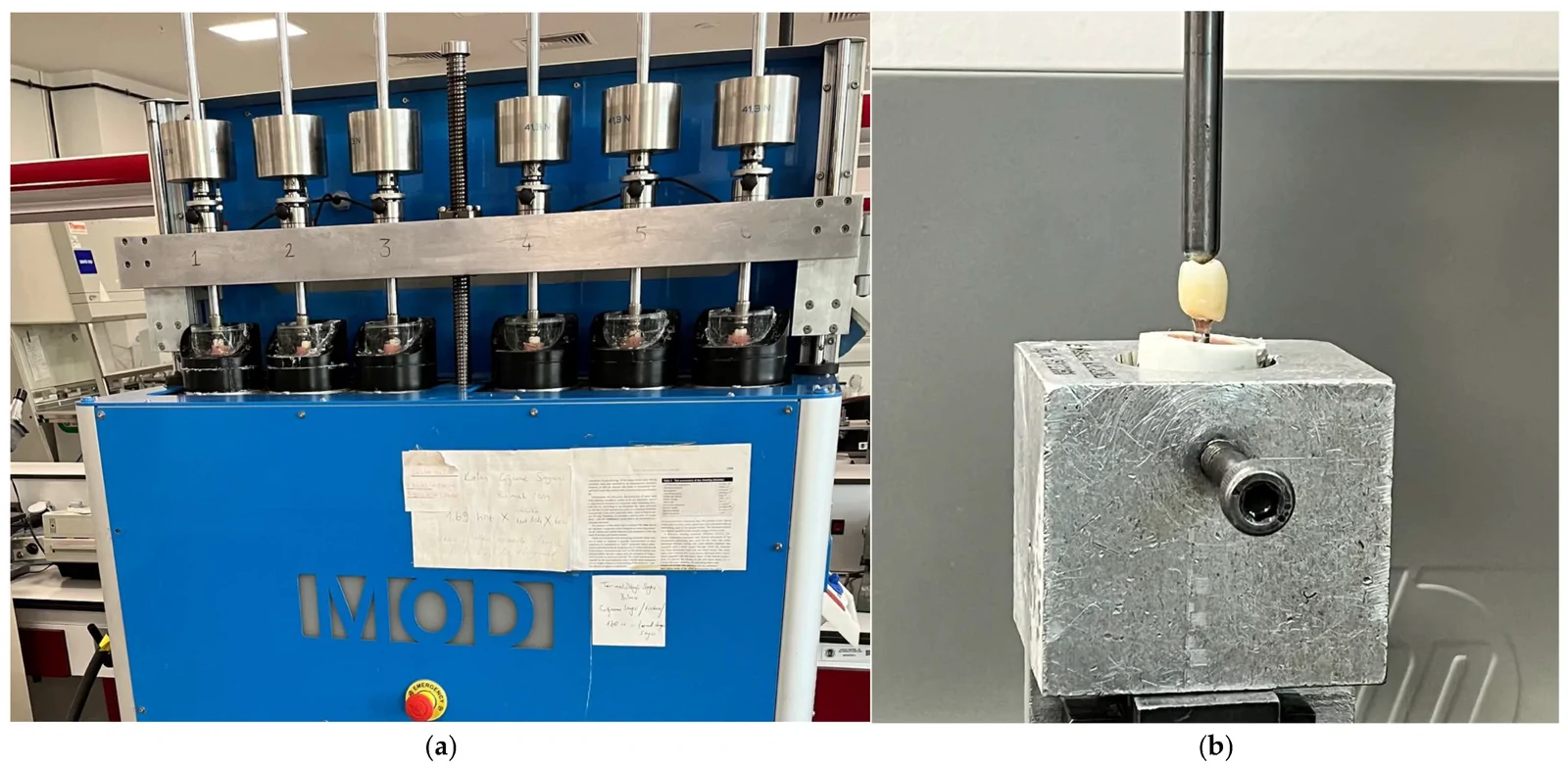
Experimental testing devices: (**a**) MOD-DENTAL chewing simulator used for thermomechanical aging; (**b**) Lloyd LRX universal testing machine used for static loading.

The same thermomechanical aging protocol was applied to all experimental groups (Figure 2a). Aging was performed in ten sequential batches, with six specimens processed simultaneously in each batch. The order in which specimens underwent aging and subsequent static loading could not be reliably reconstructed from the available records; therefore, randomization of the testing sequence could not be confirmed.

Following thermomechanical aging, all specimens were subjected to static loading using a Lloyd LRX universal testing machine, AMETEK, Hampshire, UK (Figure 2b). A 4-mm-diameter spherical loading tip was positioned on the buccal incline of the functional cusp of each mandibular first-molar crown. Vertical loading was applied at a crosshead speed of 1 mm/min.

**Figure 3:**
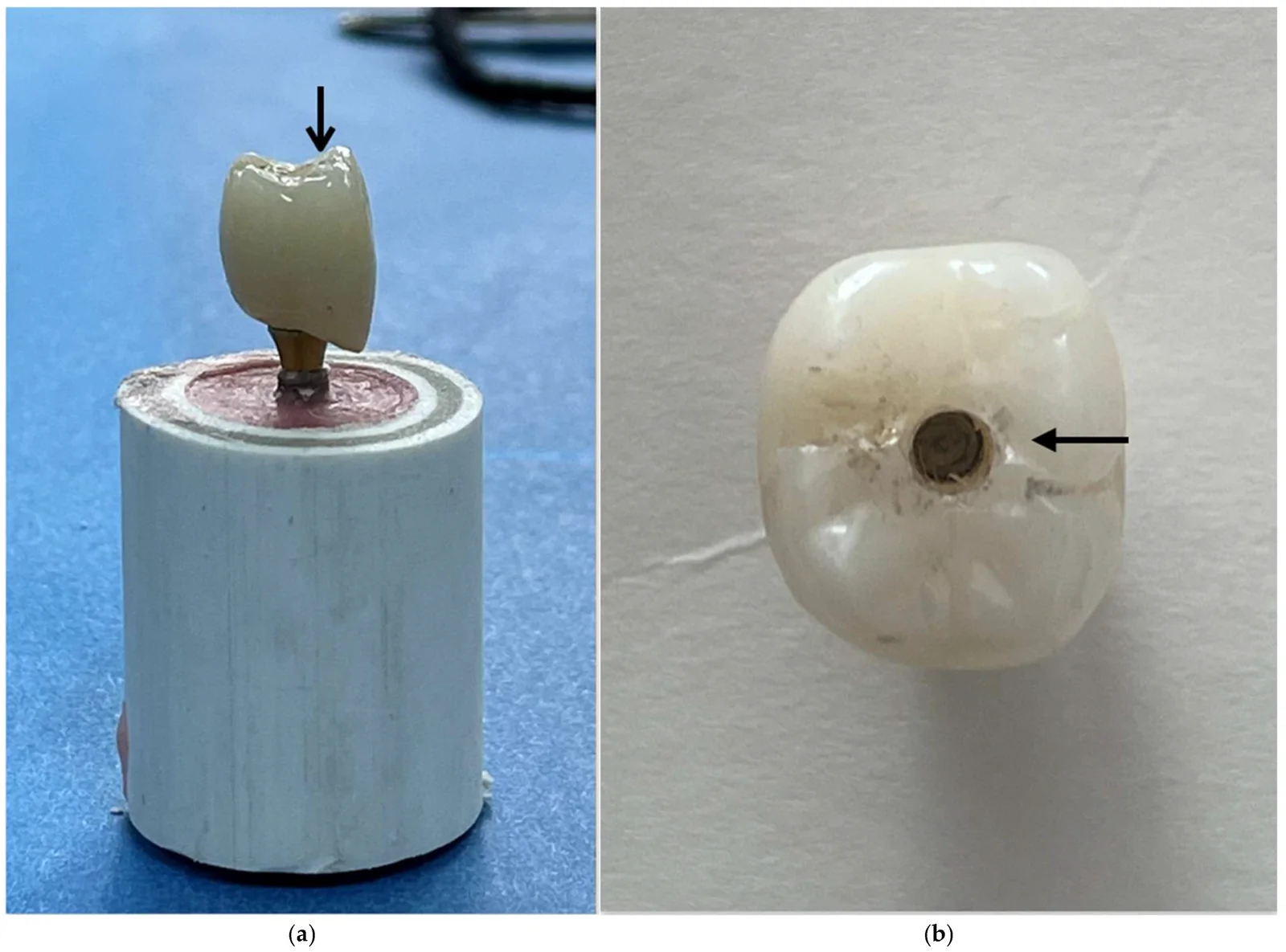
Views of the monolithic zirconia crown used in the mechanical testing protocol: (**a**) lateral view showing the loading contact region (arrow) on the buccal incline of the functional cusp; (**b**) occlusal view showing the centrally positioned screw-access channel.

To standardize the loading procedure, the same anatomical region was targeted across specimens. The centrally positioned screw-access channel, corresponding to the implant-axis reference in the common digital design, served as a visual positioning guide (Figure 3). The loading tip was securely attached to the upper assembly of the testing machine, while each specimen was fixed in a metal holding jig. Although no horizontal movement of the loading tip was programmed, relative sliding or migration of the contact point along the inclined cusp surface may have occurred as deformation progressed. Such movement was not quantitatively monitored.

During static testing, load and crosshead displacement were monitored by the testing instrument. Displacement represented crosshead travel rather than direct specimen deformation, was measured without an external extensometer, and was set to zero at the initial contact between the loading tip and the crown surface. Instrument-exported maximum recorded load values (global peak load, N) were retained for all 60 specimens, together with the available testing dates and times. These values were linked to specimen identifiers and experimental groups using the study records. However, the original continuous load–displacement recordings are no longer available for retrospective analysis.

Test termination was determined by the observed load response rather than a uniform final displacement criterion. For specimens exhibiting an abrupt load decrease associated with zirconia crown fracture, testing was terminated following the sudden drop. For specimens showing a gradual post-peak load decrease, loading continued for at least an additional 1.5 mm of crosshead travel after the decrease was observed, followed by manual termination. Consequently, final crosshead displacement varied among specimens.

The primary mechanical outcome was the maximum recorded load (global peak load, N) during static testing. This value represented the maximum load sustained by the implant-supported crown assembly under the specified loading protocol. It did not necessarily correspond to the onset of irreversible damage, which was not independently determined during testing.

Following mechanical testing and unloading, the prosthetic assemblies were disassembled, and the zirconia crowns, prosthetic screws, abutment components, and implant connections were examined separately. The original 60 specimens were assessed for macroscopic and component-level damage by visual inspection and magnified examination using a Leica stereomicroscope (Leica Microsystems, Wetzlar, Germany) at the Central Research Laboratory of Ankara University Faculty of Dentistry. The expanded component-level classification reflects more detailed reporting of post-test findings from the same specimens, without the inclusion of additional specimens or repeated mechanical tests.

For descriptive purposes, prosthetic screw deformation was distinguished from screw fracture. Deformation was defined as persistent bending or distortion without an identifiable complete discontinuity, whereas fracture was defined as a visible discontinuity of the screw. Abutment/HEX deformation and implant-connection damage were recorded as separate categories corresponding to the affected prosthetic and implant connection structures. Because multiple types of damage could occur in the same specimen, these categories were not considered mutually exclusive.

In the multi-unit configuration, the prosthetic retaining screw connecting the CAD/CAM Ti-base to the multi-unit abutment (tightened to 15 N·cm) was distinguished from the implant–multi-unit abutment connection screw (tightened to 25 N·cm). Crown–abutment separation was defined descriptively as separation at the crown–titanium component interface and was not considered, by itself, evidence of adhesive failure. Because the examinations were performed after mechanical loading, the findings could not establish the initial failure event or the chronological sequence of component damage.

Statistical analyses were performed using IBM SPSS Statistics (version 32.0; IBM Corp., Armonk, NY, USA). Normality of the model residuals was assessed using the Shapiro–Wilk test, and homogeneity of variances was evaluated using the median-centered Levene (Brown–Forsythe) test. A two-way analysis of variance (ANOVA) was conducted to examine the main effects of abutment configuration and nominal CHS, as well as their interaction, on maximum recorded load. Pairwise comparisons among the nominal CHS levels were adjusted using Tukey’s method. Effect sizes were reported as partial η^2^, and statistical significance was set at α = 0.05.

To evaluate the sensitivity of the findings to potential heterogeneity of variances, additional analyses were performed using heteroscedasticity-consistent HC3 covariance estimation within the factorial linear model. The overall CHS effect was assessed using a robust Wald F test, and pairwise marginal contrasts were adjusted for multiple comparisons using the Holm method. Games–Howell pairwise comparisons were also performed across the three nominal CHS levels, with observations pooled across abutment configurations (*n* = 20 per CHS level), to provide a complementary analysis accounting for unequal variances. Ninety-five percent confidence intervals (95% CIs) were calculated for the principal between-group comparisons. These analyses were considered supplementary sensitivity analyses to the primary two-way ANOVA.

## 3. Results

### 3.1. Maximum Recorded Load

All 60 specimens completed thermomechanical aging without failure and subsequently underwent static loading. All specimens were included in the statistical analysis. Individual maximum recorded load values and group-level summaries of component damage findings are provided in Supplementary File S1. Descriptive statistics for the six experimental groups are presented in Table 1. The highest mean maximum recorded load was observed in the Ti-base group at the lowest nominal CHS level (approximately 13.5 mm; 1961.31 ± 366.73 N), whereas the lowest mean was recorded in the multi-unit group at the intermediate nominal CHS level (approximately 15.5 mm; 1572.64 ± 162.81 N).

The Shapiro–Wilk test did not indicate a statistically significant departure from normality of the model residuals (*p* = 0.397). The median-centered Levene (Brown–Forsythe) test was also non-significant (*p* = 0.071), although this finding does not confirm homogeneity of variances. The primary two-way ANOVA showed a statistically significant main effect of nominal CHS on maximum recorded load (F(2, 54) = 4.82; *p* = 0.012; partial η^2^ = 0.152). In contrast, neither the main effect of abutment configuration (F(1, 54) = 2.39; *p* = 0.128; partial η^2^ = 0.042) nor the interaction between abutment configuration and CHS (F(2, 54) = 0.47; *p* = 0.627; partial η^2^ = 0.017) was statistically significant (Table 2).

The marginal mean maximum recorded loads, averaged across abutment configurations, were 1939.46, 1690.96, and 1646.73 N at nominal CHS levels of approximately 13.5, 15.5, and 17.5 mm, respectively. In the primary Tukey-adjusted pairwise comparisons, the lowest CHS level showed a significantly higher marginal mean maximum recorded load than the intermediate (mean difference = 248.50 N; adjusted *p* = 0.046) and highest levels (mean difference = 292.73 N; adjusted *p* = 0.016). No statistically significant difference was found between the intermediate and highest CHS levels (mean difference = 44.23 N; adjusted *p* = 0.901). However, the significance of the lowest-versus-intermediate comparison was sensitive to the analytical approach.

The group standard deviations ranged from 162.81 to 457.47 N, corresponding to a maximum-to-minimum variance ratio of approximately 7.90. To evaluate the sensitivity of the findings to unequal variances, supplementary analyses were performed. The overall CHS effect remained statistically significant in the heteroscedasticity-consistent HC3 analysis (F(2, 54) = 3.33; *p* = 0.043).

In the Games–Howell pairwise comparisons, the difference between the lowest and intermediate CHS levels was not statistically significant (mean difference = 248.50 N; 95% CI: −22.30 to 519.30 N; adjusted *p* = 0.077). In contrast, the difference between the lowest and highest CHS levels remained statistically significant (mean difference = 292.73 N; 95% CI: 28.92 to 556.54 N; adjusted *p* = 0.027). No statistically significant difference was found between the intermediate and highest levels (mean difference = 44.23 N; 95% CI: −165.94 to 254.40 N; adjusted *p* = 0.865). The Holm-adjusted HC3 marginal contrasts yielded adjusted *p*-values of 0.073, 0.043, and 0.616 for the lowest-versus-intermediate, lowest-versus-highest, and intermediate-versus-highest comparisons, respectively.

Averaged across the three nominal CHS levels, the marginal mean maximum recorded loads were 1823.21 N for the Ti-base configuration and 1694.89 N for the multi-unit configuration. Although the Ti-base configuration had a numerically higher marginal mean, the main effect of abutment configuration was not statistically significant (*p* = 0.128).

### 3.2. Failure Modes

Zirconia crown fractures were observed exclusively in the Ti-base group at the lowest nominal CHS level (approximately 13.5 mm), affecting four of ten specimens (40%; Table 3). The corresponding maximum recorded loads were 1514.98, 2128.61, 1915.78, and 1242.71 N. The remaining six specimens in this group showed metallic component damage without zirconia crown fracture, with maximum recorded loads of 2114.19, 2123.23, 2478.46, 1864.03, 2328.45, and 1902.63 N. No zirconia crown fractures were observed in the other experimental groups.

Prosthetic screw fractures were recorded only in the Ti-base groups, with frequencies of 30%, 70%, and 50% at nominal CHS levels of approximately 13.5, 15.5, and 17.5 mm, respectively. No prosthetic screw fractures were recorded in the multi-unit groups, where screw deformation was observed in 90–100% of specimens.

Abutment/HEX deformation and implant-connection damage were frequently observed in both configurations, with group-level frequencies ranging from 60% to 100% and from 90% to 100%, respectively. Crown–abutment separation was uncommon, occurring in one specimen in the Ti-base group at the lowest nominal CHS level and one specimen in the multi-unit group at the intermediate nominal CHS level (10% in each group).

**Figure 4:**
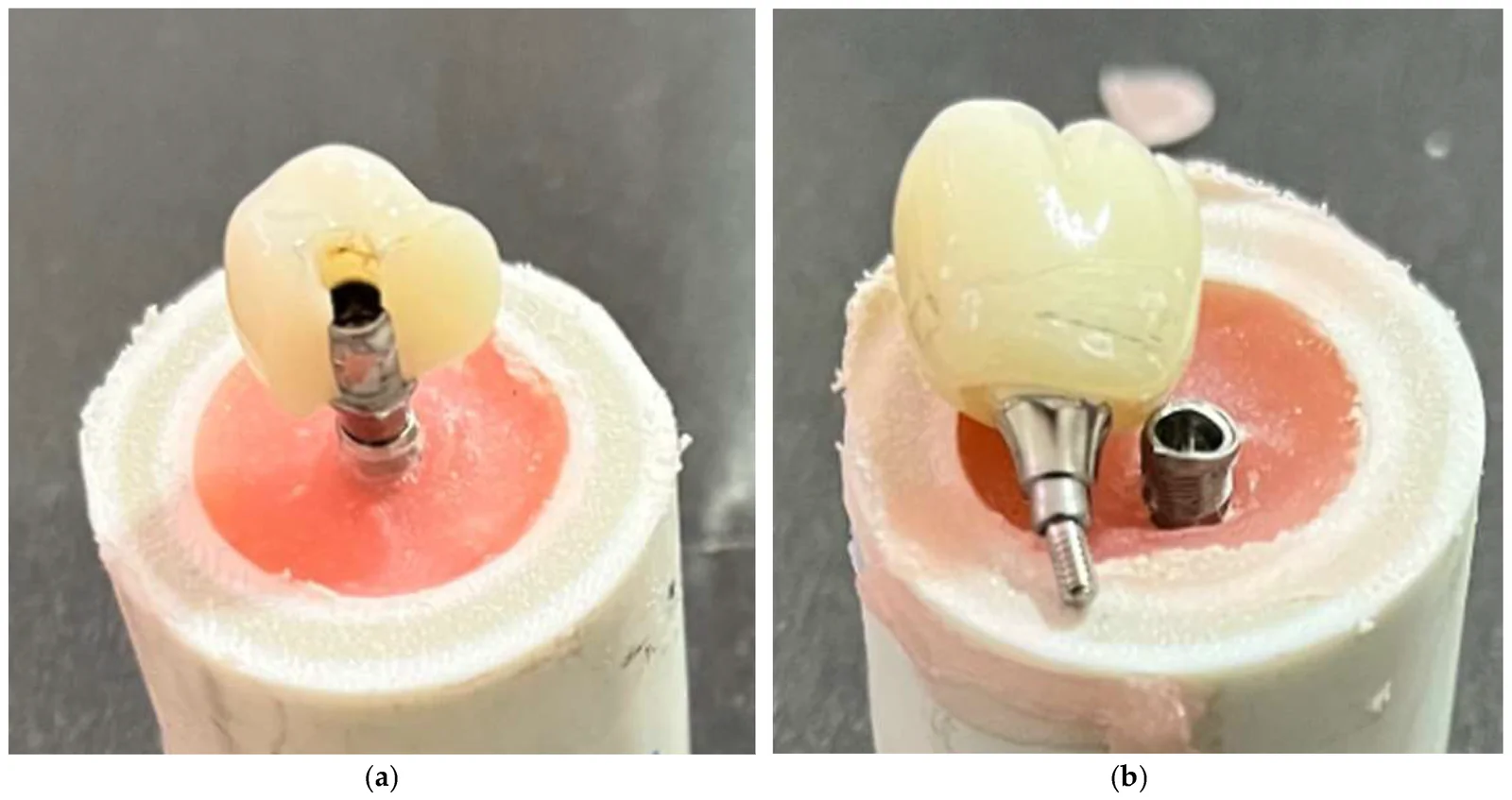

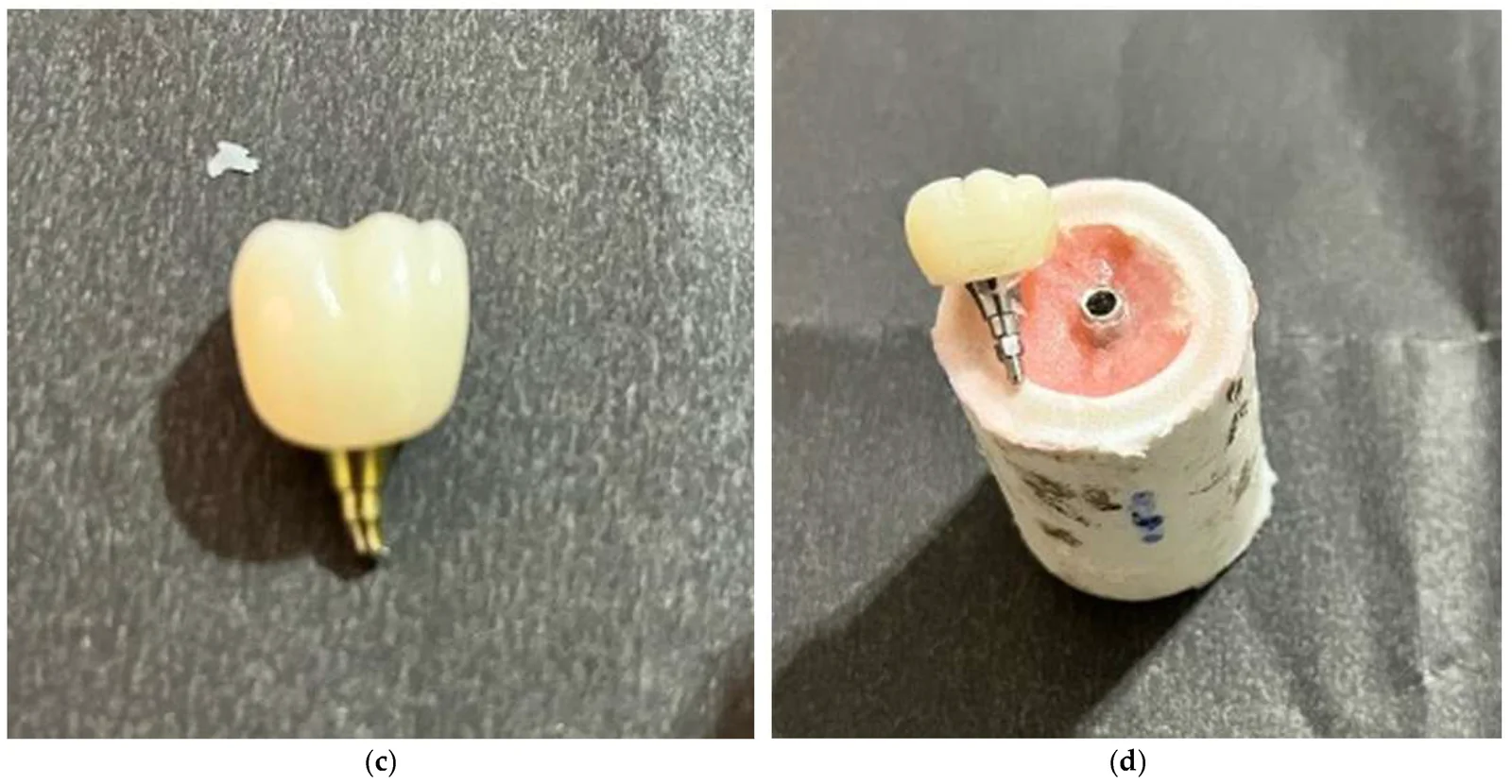
Representative post-test component-level damage following static loading: (**a**) zirconia crown fracture; (**b**) prosthetic screw deformation; (**c**) prosthetic screw fracture; and (**d**) implant-connection damage. The photographs show post-test conditions and do not establish the initiating failure event or chronological sequence of damage.

The component-level findings describe post-test damage rather than mutually exclusive primary failure events (Figure 4; Supplementary Figure S1 in Supplementary File S2). The expanded classification provides additional descriptive detail beyond the original distinction between zirconia crown fracture and permanent deformation. In particular, prosthetic screw fractures identified after loading should not be interpreted as evidence that screw fracture initiated mechanical failure. The reported damage frequencies are descriptive, and the available records do not permit reliable specimen-level linkage for all component-damage categories.

## 4. Discussion

The principal finding of this in vitro study was a significant effect of nominal crown height space (CHS) on the maximum recorded load of implant-supported monolithic zirconia crown assemblies, whereas neither abutment configuration nor the CHS × abutment configuration interaction was statistically significant. The overall CHS effect remained significant in the heteroscedasticity-consistent HC3 analysis (*p* = 0.043), although this result was close to the conventional significance threshold. The lowest-versus-highest CHS comparison remained significant in both Games–Howell and Holm-adjusted HC3 analyses, whereas the lowest-versus-intermediate comparison was significant only in the primary Tukey-adjusted analysis.

The observed effect of CHS on maximum recorded load is broadly consistent with previous experimental findings on restorative height [6,7,8]. Gehrke reported lower load-bearing capacity with increasing crown height across different implant–abutment connection designs [6], while Bagegni et al. observed lower failure loads at greater suprastructure heights [7]. However, differences in implant–prosthetic geometry, restorative materials, aging protocols, and loading conditions limit direct quantitative comparisons.

The clinical interpretation of CHS-related findings requires consideration of the different restorative-height definitions used in the literature. Within the framework described by Misch, CHS values of approximately 8–12 mm have been considered favorable for fixed implant-supported restorations, whereas values exceeding 15 mm may be associated with increased biomechanical risk [1]. Previous experimental studies have investigated crown or suprastructure heights ranging from approximately 7.5 to 18 mm [6,7,8]. However, these measurements are not necessarily equivalent to CHS as defined in the present study and should be regarded as contextual references rather than directly comparable values. Furthermore, because only three discrete nominal CHS levels were evaluated, the findings do not establish a continuous or linear relationship between CHS and maximum recorded load or identify a clinical threshold at 15 mm.

Loading conditions may influence the relationship between restorative height and mechanical performance. Previous finite element studies have suggested that increasing crown height may have relatively limited effects under axial loading but may increase stress concentrations under oblique loading [5,15,16]. An experimental study also reported no significant effect of crown height on detorque values, despite more frequent screw fractures at greater heights [17]. Because the present study used eccentric vertical loading and evaluated maximum recorded load, these findings provide biomechanical context rather than directly comparable mechanical outcomes.

Although the Ti-base groups showed numerically higher maximum recorded loads than the multi-unit groups, the main effect of abutment configuration was not statistically significant. The configurations differed in their connection architecture: the zirconia crowns were cemented directly onto implant-level Ti-bases in the Ti-base groups, whereas the multi-unit assemblies incorporated an additional abutment-level prosthetic interface. Previous studies have suggested that prosthetic configuration and connection geometry may influence the mechanical behavior of implant-supported restorations [6,7,11,12,14], although differences in experimental design limit direct comparisons. Therefore, the present findings do not establish mechanical equivalence or demonstrate the superiority of either configuration.

The primary mechanical outcome, maximum recorded load (global peak load), represented the highest load attained by the entire assembly rather than the onset of irreversible damage. In specimens exhibiting progressive metallic deformation, damage may have begun before the recorded peak, but its onset was not independently determined.

Post-test examination revealed zirconia crown fractures only in the Ti-base group at the lowest nominal CHS level, whereas damage in specimens without crown fracture predominantly involved prosthetic screws, abutment/HEX components, and implant connections. These observations describe the condition of the assemblies after testing but cannot establish the initiating failure event or the chronological sequence of component damage. Bagegni et al. similarly reported crack formation and plastic deformation in implant components, particularly at greater suprastructure heights [7], although differences in experimental conditions limit direct comparisons. These findings highlight the importance of evaluating the implant–prosthetic assembly as a whole rather than interpreting mechanical performance solely in terms of zirconia fracture resistance [8,13]. Furthermore, crown–abutment separation cannot be attributed to adhesive failure without direct examination of the bonding interface.

Several methodological limitations relate to loading geometry and its standardization. Although the loading region was visually standardized using the common digital design and centrally positioned screw-access channel, the horizontal distance between the implant axis and the actual contact point was not quantitatively measured. Potential migration of the contact point along the inclined cusp surface during loading was also not monitored. Under idealized vertical loading, the bending moment at the implant support plane depends on the applied force and horizontal contact eccentricity rather than on restoration height alone. Therefore, unmeasured variations in contact geometry may have influenced load transfer, and the observed differences in maximum recorded load cannot be attributed exclusively to CHS.

Although all restorations were designed from a common master model, geometric identity of the occlusal surfaces across CHS levels was not quantitatively verified. Restoration height was increased along the implant long axis while comparable mandibular first-molar morphology, cusp anatomy, and cusp inclinations were maintained as design targets. Nevertheless, residual geometric differences may have affected stress distribution and post-test damage patterns. Additionally, the different gingival heights of the Ti-base and multi-unit components (2.4 and 2.5 mm, respectively) resulted in a 0.1-mm difference in actual CHS between corresponding nominal groups.

Additional limitations relate to experimental loading conditions, sample size, and data availability. Eccentric vertical static loading following thermomechanical aging does not fully reproduce the multidirectional and time-dependent forces encountered during clinical function. Therefore, the findings should be interpreted as comparative mechanical responses under laboratory conditions rather than direct predictors of clinical performance. No a priori power analysis was performed, and the sample size of 10 specimens per group may have limited the detection of smaller effects, particularly those related to abutment configuration and its interaction with CHS.

Regarding data availability, the retained records include instrument-exported maximum recorded load values for all 60 specimens, available testing dates and times, study records linking the load values to specimen identifiers and experimental groups, group-level summaries of component damage, post-test photographs, and archived exocad HTML design-view files. However, the original continuous load–displacement recordings are no longer available for retrospective analysis, and complete specimen-level linkage could not be verified for all component-damage categories. Consequently, the complete loading trajectories could not be retrospectively examined, the onset of irreversible damage remained undetermined, and the interpretation of post-test damage patterns was limited.

Future research should investigate the effects of CHS across different implant diameters, implant–abutment configurations, restorative materials, and loading directions under extended cyclic loading conditions. Previous biomechanical studies suggest that implant diameter and vertical restorative dimensions may jointly influence stress distribution [18,19]. Direct assessment of stress and strain distributions using finite element analysis or experimental measurements could further clarify the mechanisms underlying component-level damage. Long-term clinical studies are also needed to determine the relevance of these laboratory findings to prosthetic complications and implant-supported restoration performance.

## 5. Conclusions

Within the limitations of this in vitro study, crown height space (CHS) significantly influenced the maximum recorded load of implant-supported monolithic zirconia crown assemblies. The overall CHS effect remained statistically significant in the HC3 sensitivity analysis (*p* = 0.043). The difference between the lowest (approximately 13.5 mm) and highest (approximately 17.5 mm) nominal CHS levels remained significant in both Games–Howell and Holm-adjusted HC3 analyses, whereas the lowest-versus-intermediate (approximately 15.5 mm) comparison depended on the statistical approach. No significant difference was detected between the intermediate and highest levels. These findings do not establish a continuous or linear decrease in maximum recorded load with increasing CHS or a clinical threshold at 15 mm.

Neither the main effect of abutment configuration nor its interaction with CHS was statistically significant, although these findings do not establish mechanical equivalence between configurations. Post-test damage predominantly involved prosthetic connection components, whereas zirconia crown fractures occurred only in the Ti-base group at the lowest nominal CHS level. The maximum recorded load did not necessarily correspond to the onset of irreversible damage, and the chronological sequence of component damage could not be determined. Therefore, the findings should be interpreted as assembly-level mechanical responses under the tested laboratory conditions rather than as direct predictors of long-term clinical performance.

## Figures and Tables

**Table 1:** Descriptive statistics for maximum recorded load according to abutment configuration and nominal crown height space (CHS), including mean ± standard deviation (SD), minimum and maximum values, and 95% confidence intervals (CIs) for the mean.

Abutment Configuration	Nominal CHS	Mean ± SD (N)	Minimum (N)	Maximum (N)	95% CI for Mean
Ti-base	≈13.5 mm	1961.31 ± 366.73	1242.71	2478.46	1698.97–2223.65
Ti-base	≈15.5 mm	1809.28 ± 336.68	1260.94	2331.78	1568.43–2050.13
Ti-base	≈17.5 mm	1699.05 ± 319.37	1294.18	2180.03	1470.58–1927.51
Multi-unit	≈13.5 mm	1917.61 ± 457.47	1357.06	2472.91	1590.36–2244.86
Multi-unit	≈15.5 mm	1572.64 ± 162.81	1359.24	1976.98	1456.17–1689.10
Multi-unit	≈17.5 mm	1594.41 ± 185.18	1212.60	1845.55	1461.94–1726.88

**Table 2:** Two-way analysis of variance for the effects of abutment configuration and nominal crown height space (CHS) on maximum recorded load.

Factor	Df	F	*p*	Partial η^2^
Abutment configuration	1	2.39	0.128	0.042
Nominal CHS	2	4.82	0.012	0.152
Abutment configuration × nominal CHS	2	0.47	0.627	0.017
Error	54	-	-	-

**Table 3:** Distribution of failure modes among the experimental groups.

Abutment Configuration	Nominal CHS	Zirconia Crown Fracture, *n* (%)	Prosthetic Screw Deformation *n* (%)	Prosthetic Screw Fracture *n* (%)	ABUTMENT/HEX Deformation *n* (%)	Implant Connection Damage *n* (%)	Crown–Abutment Separation *n* (%)
Ti-base	≈13.5 mm	4 (40)	3 (30)	3 (30)	6 (60)	10 (100)	1 (10)
Ti-base	≈15.5 mm	0 (0)	3 (30)	7 (70)	10 (100)	10 (100)	0 (0)
Ti-base	≈17.5 mm	0 (0)	5 (50)	5 (50)	10 (100)	10 (100)	0 (0)
Multi-unit	≈13.5 mm	0 (0)	10 (100)	0 (0)	10 (100)	10 (100)	0 (0)
Multi-unit	≈15.5 mm	0 (0)	9 (90)	0 (0)	9 (90)	9 (90)	1 (10)
Multi-unit	≈17.5 mm	0 (0)	10 (100)	0 (0)	10 (100)	10 (100)	0 (0)

## Data Availability

The original contributions presented in this study are included in the article/Supplementary Material. Further inquiries can be directed to the corresponding author.

## Supplementary Materials

The following supporting information can be downloaded at: https://www.mdpi.com/article/10.3390/ma19214683/s1. Supplementary File S1 contains the individual maximum recorded load values (N) for all 60 specimens and group-level summaries of component damage. Specimen-level assignments for screw damage, abutment/HEX deformation, implant-connection damage, and crown–abutment separation could not be reliably verified from the available records. Supplementary File S2 contains Supplementary Figure S1, a six-panel photographic overview of specimens following static loading across the three nominal CHS levels and two abutment configurations. Supplementary File S3 contains six archived exocad HTML design-view files representing the Ti-base and multi-unit configurations at the nominal CHS levels of approximately 13.5, 15.5, and 17.5 mm.
