# Supplementary material for: Effect of Crown Height Space and Abutment Configuration on the Maximum Recorded Load of Implant-Supported Monolithic Zirconia Crown Assemblies
Source: Materials (Basel). 2026 Nov 3;19(21):4683. doi: 10.3390/ma19214683 (PMC13764872; doi:10.3390/ma19214683)
Supplement: Supplementary file 1 [file materials-19-04683-s001.zip › Supplementary_File_S1_Data/Ozsoy_Materials_Supplementary_Statistical_Analysis.pdf]

MANUSCRIPT ID materials-4586666

Supplementary Statistical Analysis

Verification of maximum recorded loads, variance assumptions, robust sensitivity analyses and confidence intervals

Study design 2 × 3 factorial • Ti-base / Multi-unit • nominal crown height space (CHS) ≈13.5 / 15.5 / 17.5 mm • n = 60

1. Data source and analysis conventions

This report reanalyses the 60 specimen-level maximum-load observations supplied in the archived file "Minimal_Dataset_Ozsoy_et_al_UPDATED(3).xlsx". All 60 specimen IDs are unique, all load values are numeric and non-missing, and each of the six factorial cells contains 10 observations. The outcome is maximum recorded load (global peak load, N) of the complete implant-supported crown assembly, not the load at the initiation of damage.

The source workbook retains legacy design codes 8, 10 and 12. For consistency with the revised manuscript, these correspond to the nominal CHS groups ≈13.5, ≈15.5 and ≈17.5 mm, respectively; they are not themselves the final measured CHS. Actual reported CHS values are 13.4 / 15.4 / 17.4 mm for Ti-base and 13.5 / 15.5 / 17.5 mm for Multi-unit. Renaming the factor levels does not change any load value or statistical result.

Each specimen is treated as one independent experimental unit. Independence is a design assumption rather than something that can be demonstrated by statistical testing. This reanalysis verifies the supplied workbook; the individual numerical values have not been independently reconciled against original Lloyd LRX device exports.

2. Descriptive statistics

Configuration	Nominal CHS (mm)	n	Mean ± SD (N)	95% CI for mean (N)	Min–max (N)
Ti-base	≈13.5	10	1,961.31 ± 366.73	[1,698.97, 2,223.65]	1,242.71–2,478.46
Ti-base	≈15.5	10	1,809.28 ± 336.68	[1,568.43, 2,050.13]	1,260.94–2,331.78
Ti-base	≈17.5	10	1,699.05 ± 319.37	[1,470.58, 1,927.51]	1,294.18–2,180.03
Multi-unit	≈13.5	10	1,917.61 ± 457.47	[1,590.36, 2,244.86]	1,357.06–2,472.91
Multi-unit	≈15.5	10	1,572.64 ± 162.81	[1,456.17, 1,689.10]	1,359.24–1,976.98
Multi-unit	≈17.5	10	1,594.41 ± 185.18	[1,461.94, 1,726.88]	1,212.60–1,845.55

Table 1. Within-cell confidence intervals use the Student t distribution with 9 degrees of freedom. They are descriptive and not multiplicity-adjusted; factorial contrasts are provided in Section 5.

Pooled across abutment configurations (n = 20 at each nominal CHS level), the marginal means were 1,939.46 N (≈13.5 mm), 1,690.96 N (≈15.5 mm), and 1,646.73 N (≈17.5 mm). The overall Ti-base minus Multi-unit marginal mean difference was +128.33 N (classical 95% CI: −38.07 to +294.72 N).

3. Primary factorial model

A fixed-effects two-way ANOVA was fitted to the six cells, with abutment configuration (2 levels), nominal CHS (3 levels), and their interaction as categorical factors. The full factorial ordinary-least-squares model was fitted with sum-to-zero contrasts. Because the original design is balanced, classical Type-II and Type-III tests of the main effects and interaction agree.

Effect	df	SS (N^2)	MS (N^2)	F	p	Partial η^2
Abutment	1	247,012.15	247,012.15	2.391	0.1279	0.0424
Nominal CHS	2	996,002.39	498,001.19	4.820	0.0119	0.1515
Abutment × CHS	2	97,289.96	48,644.98	0.471	0.6270	0.0171
Residual	54	5,579,243.03	103,319.32	—	—	—

Table 2. Partial η^2 = SS(effect) / [SS(effect) + SS(error)]. Classical two-sided F tests; α = 0.05.

Nominal CHS showed a statistically significant overall association with the maximum recorded load in the classical factorial model (F(2,54) = 4.820; p = 0.0119). Neither the main effect of abutment configuration (p = 0.1279) nor its interaction with CHS (p = 0.6270) reached statistical significance. Non-significance does not establish equivalence or the absence of a clinically important difference.

4. Assumptions and heteroscedasticity-robust sensitivity

Residual normality was examined using Shapiro–Wilk testing of residuals from the full six-cell factorial model. Dispersion across the six experimental cells was examined using median-centered and mean-centered Levene tests, Fligner–Killeen, and Bartlett tests. These tests address different aspects of variance behavior and need not give identical p values.

Diagnostic	Test statistic	p	Interpretation
Shapiro–Wilk, residuals	W = 0.9792	0.3971	No detected residual departure from normality
Brown–Forsythe (median-centered Levene)	F(5,54) = 2.171	0.0709	Non-significant; not evidence of equal variances
Mean-centered Levene	F(5,54) = 4.976	0.0008	Evidence of heterogeneity
Fligner–Killeen	$\chi^2(5) = 11.300$	0.0458	Evidence of heterogeneity
Bartlett	$\chi^2(5) = 12.199$	0.0322	Evidence of heterogeneity; normality-sensitive

The largest versus smallest observed within-cell standard deviations were 457.47 N and 162.81 N; the ratio of their variances was 7.90. Given this heterogeneity, classical ANOVA inference was supplemented by heteroscedasticity-consistent HC3 covariance estimation.

Specified HC3 contrasts and an alternative convention

The principal heteroscedasticity-robust sensitivity check uses the full factorial model with sum-to-zero coding and Type-III Wald F tests based on the HC3 covariance matrix. This tests equality of equally weighted, abutment-averaged CHS marginal means. Denominator degrees of freedom are 54. In this formulation the CHS effect remains significant (F(2,54) = 3.328, p = 0.0434).

Effect	Classical p	HC3 Type-III F	HC3 Type-III p	HC3 Type-II p*
Abutment	0.1279	2.152	0.1482	0.0682
Nominal CHS	0.0119	3.328	0.0434	0.0510
Interaction	0.6270	0.457	0.6354	0.6354

Table 4. *An alternative HC3 Type-II ANOVA convention yields CHS p = 0.0510; it is not the same marginal-hypothesis test as the Type-III Wald test. This analysis-method sensitivity should be considered when interpreting borderline p values.

5. Pairwise CHS contrasts and confidence intervals

CHS comparisons are based on marginal means averaged equally across Ti-base and Multi-unit. For classical comparisons, Tukey familywise adjustment was applied using the factorial residual mean square (df = 54). As sensitivity analyses, Games–Howell was applied to the three pooled CHS levels (n = 20 per level, permitting unequal pooled variances), and three HC3 marginal-mean contrasts from the factorial model were adjusted using Holm's method. The pooled Games–Howell analysis does not replace factorial adjustment for abutment configuration.

Nominal CHS contrast (mm)	Mean Δ (N)	Tukey 95% CI (N)	Tukey p	Games–Howell 95% CI (N)	GH p	HC3 + Holm p
≈13.5 vs ≈15.5	+248.50	[3.53, 493.46]	0.0461	[−22.30, 519.30]	0.0773	0.0731
≈13.5 vs ≈17.5	+292.73	[47.77, 537.70]	0.0155	[28.92, 556.54]	0.0270	0.0425
≈15.5 vs ≈17.5	+44.23	[−200.73, 289.20]	0.9011	[−165.94, 254.40]	0.8653	0.6156

Table 5. Positive Δ indicates greater mean maximum recorded load at the lower nominal CHS. Tukey and Games–Howell 95% CIs account for three pairwise comparisons within each procedure. HC3 + Holm values are adjusted p values; simultaneous HC3 confidence intervals are not claimed here.

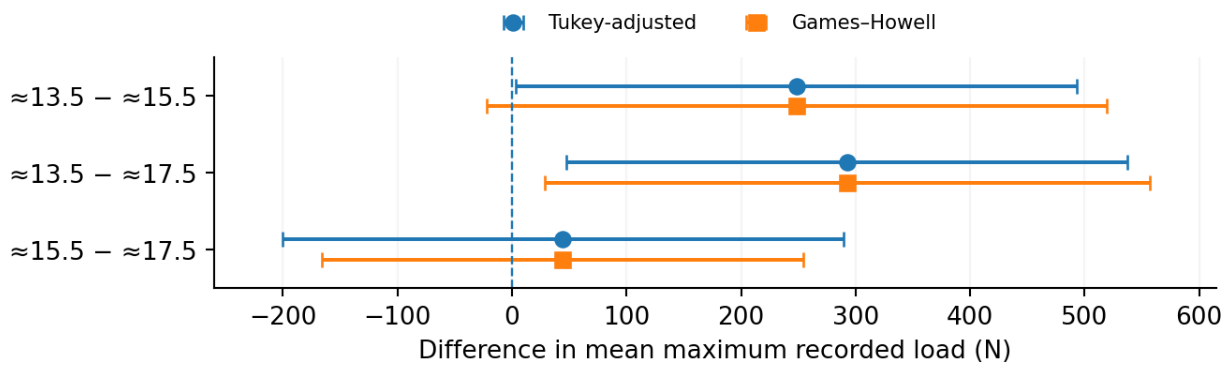

Figure 1. Pairwise mean differences and familywise-adjusted 95% confidence intervals. Intervals crossing zero are not significant by the corresponding procedure at α = 0.05.

The ≈13.5 versus ≈17.5 mm contrast was statistically significant under Tukey ($p = 0.0155$), Games–Howell ($p = 0.0270$), and HC3 + Holm ($p = 0.0425$). The ≈13.5 versus ≈15.5 mm contrast was significant only under classical Tukey ($p = 0.0461$), not under Games–Howell ($p = 0.0773$) or HC3 + Holm ($p = 0.0731$). No significant ≈15.5 versus ≈17.5 mm difference was detected by any of the three methods.

6. Observation-level influence and failure data linkage

Influence statistics were computed from the factorial model. The largest Cook's distance was 0.1028 (specimen ID 9, Ti-base, nominal CHS ≈13.5 mm; peak load 1,242.71 N); its externally studentized residual was −2.46. This observation is flagged for source-record inspection, not excluded. Type-III classical leave-one-out reanalysis (59 observations per iteration; sum contrasts) produced p ranges of 0.0026–0.0277 for CHS, 0.0584–0.1945 for abutment, and 0.4300–0.8011 for the interaction. No single deletion changed which of these omnibus effects crossed $p = 0.05$.

The supplied specimen-level workbook uses a simplified primary failure label: four observations are classified as zirconia crown fracture (IDs 1, 3, 8, 9; all Ti-base, nominal CHS ≈13.5 mm), and 56 as "prosthetic component deformation." This single-label field cannot establish that screw fracture, abutment/HEX deformation, implant connection damage, or crown–abutment separation was absent. Such component-level findings may overlap and require independent, specimen-linked examination records. Accordingly, no component-specific inferential claims are based on the simplified labels in this report.

7. Reconciliation of historical summary discrepancies

The latest archived specimen-level values yield the means and SDs reported in Table 1. Two means from an earlier manuscript iteration differ slightly from these recalculations:

Configuration / nominal CHS	Earlier mean (N)	Current mean (N)	Change (N)	Earlier / current SD (N)
Ti-base / ≈17.5 mm	1,698.97	1,699.05	+0.08	319.34 / 319.37
Multi-unit / ≈15.5 mm	1,572.50	1,572.64	+0.14	162.77 / 162.81

The current 10 Multi-unit / ≈15.5 mm specimen values sum to 15,726.357722 N, yielding a mean of 1,572.635772 N (rounded: 1,572.64 N). The current 10 Ti-base / ≈17.5 mm values yield a mean of 1,699.046210 N (rounded: 1,699.05 N). The historical means cannot both be explained as alternative two-decimal rounding of these same values. The archived dataset alone does not identify the reason for the earlier values; attribution to transcription, extraction or rounding error would therefore be speculative. Independent comparison with the historical analysis file and original device exports is required to establish the provenance of the change.

8. Interpretation, reporting and reproducibility

The factorial analysis supports an overall association between nominal CHS and maximum recorded load, but the robustness of the inference depends in part on how heteroscedastic main-effect contrasts are defined (HC3 Type-III $p = 0.0434$; alternative HC3 Type-II $p = 0.0510$). The ≈13.5 versus ≈17.5 mm pairwise difference was the most method-consistent finding. Evidence for the ≈13.5 versus ≈15.5 mm difference is sensitive to variance assumptions and multiplicity adjustment. Neither a non-significant abutment effect nor a non-significant interaction should be presented as proof of equivalence. The observed associations should not be attributed to CHS alone without accounting for unquantified loading geometry and possible differences in component-level response.

Calculations were reproduced using Python 3, NumPy, SciPy 1.17.0 and statsmodels 0.14.6. Model: ordinary least squares with full factorial categorical effects and sum contrasts; classical F tests use 54 denominator degrees of freedom. Robust HC3 joint tests use the same full model with the HC3 sandwich covariance. Games–Howell comparisons use Welch–Satterthwaite degrees of freedom for each pair; HC3 pairwise p values are adjusted by Holm across the three marginal CHS contrasts. Descriptive means, SDs and CIs were calculated from unrounded specimen-level values. No retrospective observed-power calculation was performed.

Data-integrity qualification. This statistical report verifies the provided numerical dataset. Before final journal submission, link the detailed component-level damage observations to specimen IDs in the separate supplementary dataset, and confirm the two historical mean discrepancies against original source records. Neither issue can be resolved by statistical recalculation alone.

Appendix A. Source-level specimen linkage (archived broad classification)

The following audit index reproduces all 60 specimen IDs, maximum recorded loads and the failure label stored in the uploaded source workbook. It is not a substitute for verified, component-level damage observations.

ID-to-group key: 1–10, Ti-base / ≈13.5 mm; 11–20, Ti-base / ≈15.5 mm; 21–30, Multi-unit / ≈17.5 mm; 31–40, Multi-unit / ≈13.5 mm; 41–50, Multi-unit / ≈15.5 mm; 51–60, Ti-base / ≈17.5 mm.

ID	Peak N	Mode	ID	Peak N	Mode	ID	Peak N	Mode
1	1,514.98	ZCF	21	1,212.60	PCD*	41	1,976.98	PCD*
2	2,114.19	PCD*	22	1,577.91	PCD*	42	1,600.77	PCD*
3	2,128.61	ZCF	23	1,624.80	PCD*	43	1,516.73	PCD*
4	2,123.23	PCD*	24	1,557.82	PCD*	44	1,580.88	PCD*
5	2,478.46	PCD*	25	1,668.65	PCD*	45	1,642.58	PCD*
6	1,864.03	PCD*	26	1,711.78	PCD*	46	1,549.09	PCD*
7	2,328.45	PCD*	27	1,845.55	PCD*	47	1,477.46	PCD*
8	1,915.78	ZCF	28	1,365.90	PCD*	48	1,359.24	PCD*
9	1,242.71	ZCF	29	1,627.71	PCD*	49	1,469.39	PCD*
10	1,902.63	PCD*	30	1,751.37	PCD*	50	1,553.24	PCD*
11	1,820.17	PCD*	31	1,357.06	PCD*	51	1,437.72	PCD*
12	1,373.79	PCD*	32	1,532.19	PCD*	52	1,675.51	PCD*
13	2,031.28	PCD*	33	1,683.77	PCD*	53	1,596.01	PCD*
14	2,011.84	PCD*	34	1,601.87	PCD*	54	2,092.11	PCD*
15	1,529.25	PCD*	35	2,414.52	PCD*	55	2,019.28	PCD*
16	1,260.94	PCD*	36	2,468.76	PCD*	56	1,294.18	PCD*
17	1,990.41	PCD*	37	2,397.35	PCD*	57	1,578.32	PCD*
18	2,025.68	PCD*	38	1,671.55	PCD*	58	1,815.77	PCD*
19	2,331.78	PCD*	39	2,472.91	PCD*	59	2,180.03	PCD*
20	1,717.70	PCD*	40	1,576.16	PCD*	60	1,301.54	PCD*

ZCF: recorded zirconia crown fracture. PCD*: source workbook's simplified category "prosthetic component deformation." This label does not rule out additional screw, abutment, implant-connection or separation findings. Source values are displayed at two decimals; analyses use all stored decimal places.
