# Supplementary material for: Effect of Crown Height Space and Abutment Configuration on the Maximum Recorded Load of Implant-Supported Monolithic Zirconia Crown Assemblies
Source: Materials (Basel). 2026 Nov 3;19(21):4683. doi: 10.3390/ma19214683 (PMC13764872; doi:10.3390/ma19214683)
Supplement: Supplementary file 1 [file materials-19-04683-s001.zip › Supplementary_File_S2_PostTest_Images/Supplementary_Figure_S1_Word.pdf]

Supplementary Figure S1

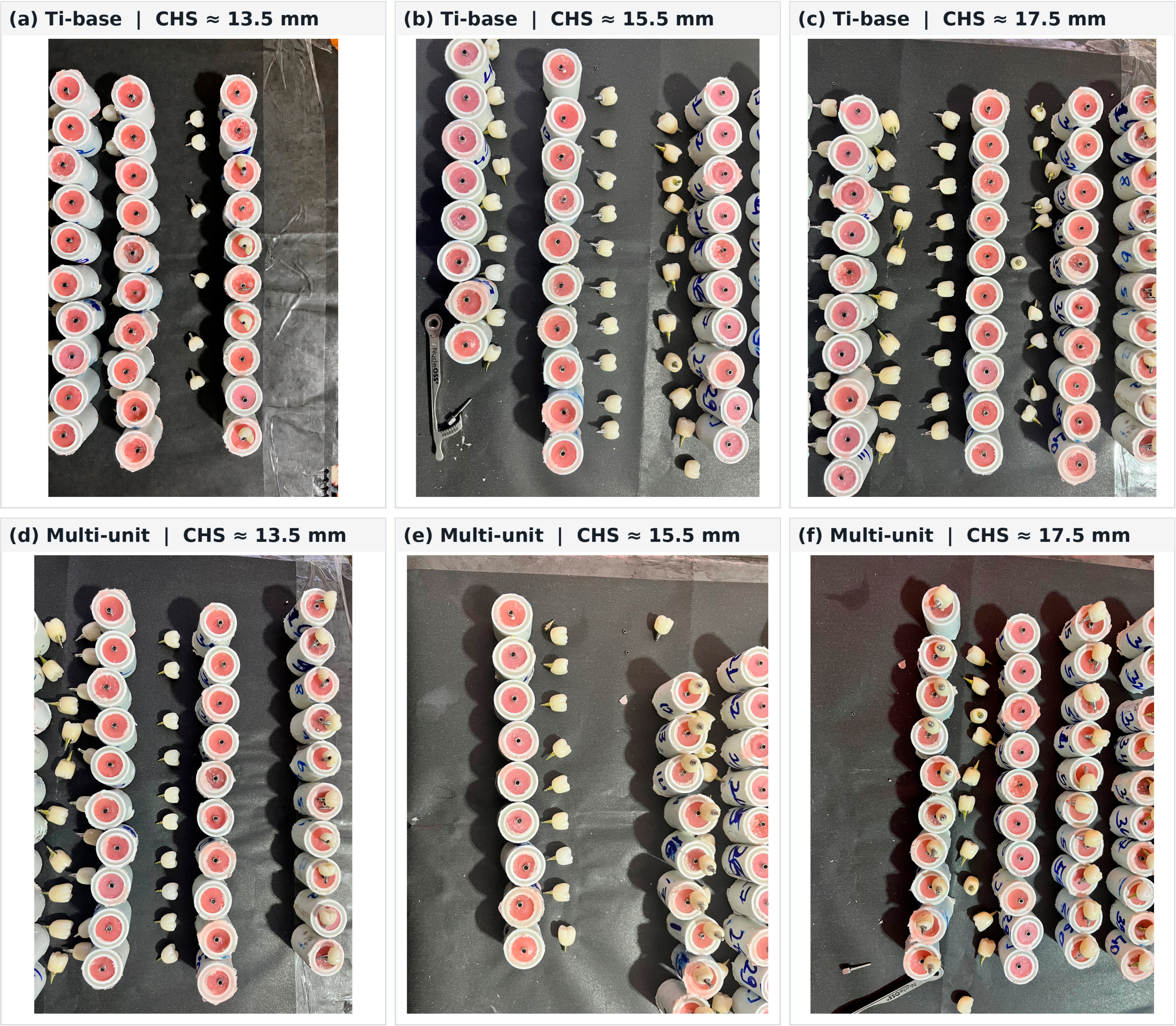

Photographic fields may include adjacent specimens near panel margins.

Supplementary Figure S1. Photographic overview of the experimental specimens following static loading. Panels (a–c) show the Ti-base configurations at nominal crown height space (CHS) levels of approximately 13.5, 15.5, and 17.5 mm, respectively. Panels (d–f) show the corresponding Multi-unit configurations. The photographs document the post-test appearance of implant-supported crown assemblies and separated prosthetic components. Adjacent specimen series may be visible near the panel margins. These images are supplementary descriptive documentation and do not independently confirm individual damage assignments or establish the initiation or sequence of component-level damage.
