# Supplementary material for: Effect of Crown Height Space and Abutment Configuration on the Maximum Recorded Load of Implant-Supported Monolithic Zirconia Crown Assemblies
Source: Materials (Basel). 2026 Nov 3;19(21):4683. doi: 10.3390/ma19214683 (PMC13764872; doi:10.3390/ma19214683)
Supplement: Supplementary file 1 [file materials-19-04683-s001.zip › Supplementary_File_S3_exocad_Designs/MultiUnit_CHS_13p5mm.html]

exocad webview


JavaScript is not enabled in this browser!

Your current HTML browser does not support JavaScript. Please use Chrome, Firefox or supported browsers, or use the exocad webview app from Android or iOS store to open the HTML file.

exocad webview - Version 1.6.11  
Copyright © 2023 exocad GmbH
